# Childhood aplastic anaemia with paroxysmal nocturnal haemoglobinuria clones: A retrospective single-centre study in South Africa

**DOI:** 10.4102/ajlm.v11i1.1537

**Published:** 2022-06-06

**Authors:** Candice L. Hendricks, Ashen Naidoo, Rajendra Thejpal, Nadine Rapiti, Beverley Neethling, Yasmin Goga, Suvarna Buldeo

**Affiliations:** 1 Department of Paediatric Haematology, Faculty of Health Sciences, School of Medicine, University of KwaZulu-Natal, Durban, South Africa; 2 Department of Paediatric Haematology, Inkosi Albert Luthuli Central Hospital, Durban, South Africa; 3 Department of Haematology, Faculty of Health Sciences, University of KwaZulu-Natal, Durban, South Africa; 4 National Health Laboratory Service, Inkosi Albert Luthuli Central Hospital, Durban, South Africa

**Keywords:** paroxysmal nocturnal haemoglobinuria clones, aplastic anaemia, paediatrics, flow cytometry, HLA typing

## Abstract

**Background:**

Paroxysmal nocturnal haemoglobinuria (PNH) clones in children are rare but commonly associated with aplastic anaemia (AA) and myelodysplasia.

**Objective:**

This study aimed to determine the prevalence of PNH clones in paediatric patients with idiopathic AA, identify differences in clinical and laboratory features and outcomes, and determine the impact of clone size on clinical presentation.

**Methods:**

Patients with confirmed idiopathic AA who were tested for PNH between September 2013 and January 2018 at the Inkosi Albert Luthuli Central Hospital, Durban, KwaZulu-Natal, South Africa, were included. PNH clones were detected in neutrophils and monocytes by flow cytometry using fluorescent aerolysin, CD24, CD66b and CD14.

**Results:**

Twenty-nine children with AA were identified and 11 were excluded. Ten patients (10/18, 55.6%) had PNH clones ranging from 0.11% to 24%. Compared to the PNH-negative group, these children were older (median: 10 years vs 4 years, *p* = 0.02) and had significantly lower total white cell counts (median 1.7 × 10^9^/L vs 3.2 × 10^9^/L; *p* = 0.04). There was no difference in median absolute neutrophil count or haemoglobin concentration. Four patients in each group received immunosuppressive therapy (IST). At six months, all four patients with PNH clones had responded, compared to one in the PNH-negative group.

**Conclusion:**

More than half of children with AA had a PNH clone. The size of the clone did not impact clinical severity; however, IST use may positively impact prognosis. We recommend early initiation of IST in patients with AA to avoid delays associated with human leukocyte antigen typing.

## Introduction

Paroxysmal nocturnal haemoglobinuria (PNH) is an acquired haematopoietic stem cell disorder.^1^ It is rare in children and results from a somatic mutation in the glycophosphatidylinositol (GPI) glycan A gene on the short arm of the X chromosome.^1,2,3^ The GPI glycan A gene encodes GPI-anchored markers, including CD55 and CD59, which protect cells from complement-mediated attack.^4,5^ Although the condition is classically described as presenting with nocturnal haemoglobinuria, this finding is not generally common and is particularly rare in children.^4,6^

Patients may also present with bone marrow failure, and the presence of PNH clones has been shown in a large percentage of children with aplastic anaemia (AA).^2,7^ This association was described by Ware et al. in 1991,^8^ where the largest initial paediatric PNH cohort was found to have features very different from their adult counterparts. Since PNH clones are often found in AA, and patients with clinical PNH often progress to AA,^9^ what is the theory of association? The prevailing theory is that haematopoietic stem cells harbouring the PNH clone are resistant to the immune-mediated damage that occurs during AA and this promotes the expansion of these ‘unaffected’ cells,^5,10^ also termed the “escape mechanism”. According to Luzatto,^10^ the progression of AA to PNH is thus “the rule rather than the exception”. Very little knowledge exists on the difference in clinical presentation and response to treatment in children with and without PNH clones, particularly on the African continent. Though AA is the most common presentation, there have been case series where thromboses and haemoglobinuria are documented.^4^

Flow cytometry-based tests are the most accurate tests for the detection of PNH clones.^7,11^ Classically, granulocytes and monocytes are analysed to determine the absence of GPI-linked proteins.^3^ Red blood cells are not thought to represent the true size of the clone, as ongoing haemolysis and the presence of transfused cells usually confound results. Paroxysmal nocturnal haemoglobinuria clone sizes < 1% require validation within each laboratory to determine accuracy. These smaller clone sizes are, however, becoming more important to identify, as their presence allows for future monitoring of clone size and the potential development of classic PNH.^2^ A previous study described the 10-year probability of developing PNH disease from a PNH clone to be 10.2%.^2^

Historically, patients with AA were managed either supportively with transfusions or cured with a haematopoietic stem cell transplant (HSCT).^12,13^ Before immunosuppressive therapy (IST) became available, early reports from Africa showed very high mortality rates in symptomatically treated patients.^14,15^ According to the North American Pediatric Aplastic Anemia Consortium, most centres would only opt for HSCT as first-line therapy in patients presenting with acquired severe AA and who have a matched sibling donor.^16^ In the absence of a sibling donor, IST with anti-thymocyte globulin (ATG) and cyclosporin becomes the best option.^17,18^ Haematopoietic stem cell transplant from a matched unrelated-donor is only recommended for patients who do not respond to first-line IST.

In our setting, the majority of patients are of recent African ancestry, where significant human leukocyte antigen (HLA) diversity exists.^19,20^ All patients diagnosed with AA in our unit routinely have HLA typing performed. If siblings from the same parents are present, they too are tested, and if they are a match, patients are referred for HSCT. A donor search for local (South African) donors is also conducted via the South African bone marrow registry. Uninsured patients treated within the state/public health system can be transplanted within the state/public sector if there is a compatible unrelated local donor identified from the registry. Insured patients can in some instances access compatible international donors. Human leukocyte antigen typing and the subsequent registry searches to find matches take time, and there is a low likelihood of finding an HLA match for transplantation. This prolongs symptomatic treatment with the risk of multiple transfusion exposures.

Our study was conducted at a quaternary hospital in Durban, South Africa. The haematology laboratory provides in-house flow cytometry services and has been using the fluorescent aerolysin method to detect the presence of PNH clones in patients since September 2013.

This study aimed to determine the prevalence of PNH clones in paediatric patients with AA, identify differences in clinical and laboratory features and treatment responses between patients with and without PNH clones, and determine the impact of clone size on clinical presentation.

## Methods

### Ethical considerations

Ethics approval was obtained from the Biomedical Research Ethics Committee of the University of KwaZulu-Natal (BCA325/18). Individual patient consent was not required due to the study being retrospective. Data were password-protected, and patient confidentiality was maintained by only including patient hospital numbers on the final datasheets.

### Study population

The electronic database of the Paediatric Haematology-Oncology unit of the Inkosi Albert Luthuli Central Hospital, Durban, South Africa, was screened to identify patients with a diagnosis of AA between 01 September 2013 and 31 January 2018. The diagnosis was based on pancytopenia secondary to bone marrow hypoplasia in the absence of a clonal malignant disorder. Patients who had an inherited bone marrow failure syndrome such as Fanconi anaemia, or who were not tested for PNH, were excluded. The electronic medical files of these children were searched on the hospital information system (Meditech, Westwood, Massachusetts, United States) as well as the laboratory information system (Trak Care, Intersystems, Cambridge, Massachusetts, United States) to retrieve demographic and clinical information, as well as laboratory test results. Patients with and without a PNH clone were compared with respect to demographics, laboratory parameters, presenting complaints, management and outcomes. The severity of AA was determined based on Camitta’s criteria (cited by Marsh et al.).^21^ Haematuria was measured using a urine dipstick and therefore was not distinguished from haemoglobinuria. Immunosuppressive therapy was administered in the form of rabbit or horse ATG, where appropriate. All patients received premedication with antihistamines and antipyretics prior to the daily infusion, as well as intravenous methylprednisone daily during the administration of the drug course, followed by a course of oral prednisone and cyclosporin. Response to IST was monitored at 6 months and 1-year post-administration. A response was deemed complete if full blood count parameters normalised (absolute neutrophil count [ANC] ≥ 1.5 × 10^9^/L, haemoglobin ≥ 11 g/dL, and platelet count ≥ 100 × 10^9^/L), partial if there was transfusion independence in the presence of cytopenias, and none if there was continued transfusion dependence.^22^

### Laboratory analysis

**FIGURE 1:**
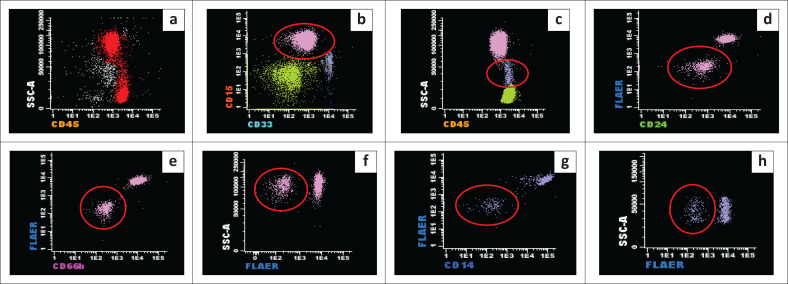
Gating strategy for paroxysmal nocturnal haemoglobinuria clones, South Africa, September 2013 – January 2018. (a) All white blood cells gated using CD45; (b) gated neutrophils; (c) gated monocytes; (d, e, f) the dual-negative granulocytes; (g, h) dual-negative monocytes.

Flow cytometry (FacsCanto II, Becton Dickinson, San Jose, California, United States) was performed to detect PNH clones using non-GPI-linked markers (CD15 and CD33) to identify the monocytes and neutrophils, and GPI-linked markers (fluorescent aerolysin, CD24, CD66b and CD14) to detect the loss of GPI anchors in a sequential gating strategy (Figure 1). After performing doublet discrimination using forward scatter-area vs forward scatter-height and removing debris, all white blood cells were gated using CD45. Neutrophils were then discriminated by gating on CD15-positive, CD33-dim and high-side scatter white blood cells. Monocytes were discriminated by gating on CD33-bright, CD15-negative and low-side scatter white blood cells. Neutrophil PNH clones were defined as clones negative for CD24, CD66b and fluorescent aerolysin, and monocyte PNH clones were clones negative for CD14 and fluorescent aerolysin.

A loss of two GPI-linked markers on both the monocyte and neutrophil lineage was required to detect a clone. All clone sizes were included as per the latest guidelines of the International Clinical Cytometry Society/European Society for Clinical Cell Analysis,^23^ which classify clones as “PNH clone” for PNH populations > 1.0%, “minor population of PNH cells” or “minor PNH clone” for PNH populations between 0.1% and 1.0%, and “rare cells with GPI deficiency” or “rare cells with PNH phenotype” for PNH populations < 0.1%. For uniformity, we refer to all PNH populations, even if < 1.0%, as clones in this publication.

### Data analysis

Statistical analysis was performed using GraphPad Prism version 8.4.3 (2020, San Diego, California, United States). A multiple t-test which was used to determine the statistical difference between numerical variables using means (Holm-Sidak method) and a chi-square test or Fisher’s exact test which was used for categorical variables. A *p* < 0.05 was considered statistically significant.

## Results

**FIGURE 2:**
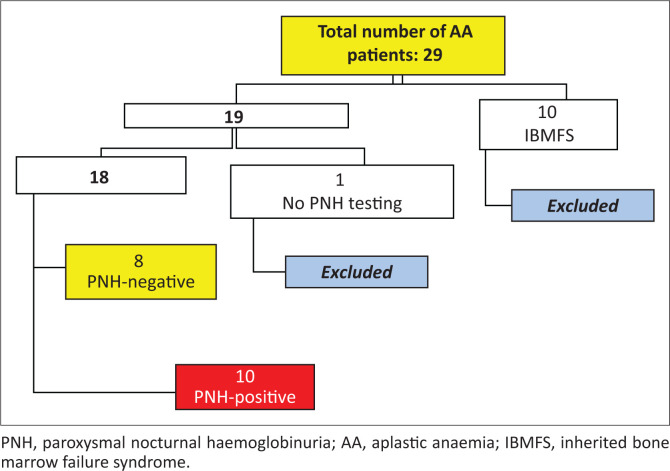
Study inclusion and exclusion criteria for patients with aplastic anaemia at the Inkosi Albert Luthuli Central Hospital, Durban, South Africa, September 2013 – January 2018.

Twenty-nine patients were diagnosed with AA during the study period, ten of whom had Fanconi anaemia and were subsequently excluded (Figure 2). Of the remaining 19 patients, only one patient did not have PNH testing performed. Eighteen patients were thus included. Ten patients had a PNH clone (10/18; 55.6%); six of which were classified as having a “PNH clone” and four as having a “minor population of PNH cells”.

Patients with a PNH clone were older compared to those without the clone (median age: 10 years vs 4 years; *p* = 0.02) (Table 1). Female patients made up the majority of patients in both the PNH-negative group (5/8; 62.5%) and the PNH-positive group (6/10; 60.0%). The full blood count results showed no difference in haemoglobin concentration and platelet count between the two groups. Paroxysmal nocturnal haemoglobinuria-positive patients had a significantly lower median total white cell count of 1.7 × 10^9^/L compared to 3.2 × 10^9^/L in PNH-negative patients (*p* = 0.04). However, the median ANC was similar in both groups: 0.26 × 10^9^/L in the PNH-positive group and 0.32 × 10^9^/L in the PNH-negative group. Lactate dehydrogenase results were known in 80% (8/10) of PNH-positive and 50% (5/10) of PNH-negative patients and all results were within the normal range. All patients in the PNH-positive and PNH-negative groups had complete information relating to the reticulocyte production index, which was low in all patients. Hypocellularity was also confirmed in all patients in both groups by bone marrow aspiration and trephine biopsy.

**TABLE 1:** Clinical characteristics of paroxysmal nocturnal haemoglobinuria-positive and -negative patients at the Inkosi Albert Luthuli Central Hospital, Durban, South Africa, September 2013 – January 2018.

Patient characteristics	PNH +					PNH –					*p*
	*n*	%	Median	Mean	Range	*n*	%	Median	Mean	Range	
Number of patients	10	55.6	-	-	-	8	44.4	-	-	-	-
**Demographic information**											
Median age in years	-	-	10.00	-	-	-	-	4.00	-	-	**0.016**
Male patients	4	40.0	-	-	-	3	37.5	-	-	-	0.914
Female patients	6	60.0	-	-	-	5	62.5	-	-	-	-
**Full blood count variables at diagnosis**											-
Haemoglobin concentration (g/dL)	-	-	7.15	6.97	2.20–12.70	-	-	7.15	7.05	4.30–9.00	0.996
Platelet count ×10^9^/L	-	-	7.50	24.00	1.00–88.00	-	-	22.50	25.00	2.00–54.00	0.996
WCC (×10^9^/L)	-	-	1.70	1.98	1.20–4.08	-	-	3.20	3.06	1.80–3.70	**0.040**
ANC (×10^9^/L)	-	-	0.26	0.28	0.02–0.51	-	-	0.32	0.42	0.01–1.20	0.765
Reticulocyte production index	-	-	0.10	0.16	0.00–0.40	-	-	0.15	0.14	0.00-0.30	0.981
Lactate dehydrogenase (U/L)	-	-	239.00	229.50	192.00–308.00	-	-	299.50	315.50	206.00–457.00	0.440
**Severity of aplastic anaemia**											
Non-severe	1	10.0	-	-	-	2	25.0	-	-	-	0.818
Severe	6	60.0	-	-	-	3	37.5	-	-	-	-
Very severe	3	30.0	-	-	-	3	37.5	-	-	-	-
**Bone marrow cellularity**											
Hypocellular	10	100.0	-	-	-	8	100.0	-	-	-	N/A
**Clinical presentation**											
Haematuria	0	0.0	-	-	-	2	25.0	-	-	-	N/A
Bleeding	7	70.0	-	-	-	7	87.5	-	-	-	-
Thromboses	0	0.0	-	-	-	0	0.0	-	-	-	-
**Treatment**					-					-	
IST	4	40.0	-	-	-	4	50.0	-	-	-	> 0.999
Symptomatic	5	50.0	-	-	-	4	50.0	-	-	-	
Transplant	1	10.0	-	-	-	0	0.0	-	-	-	N/A
**Outcome**											
Deceased	5	50.0	-	-	-	3	37.5	-	-	-	> 0.999
Alive	5	50.0	-	-	-	3	37.5	-	-	-	-
**Lost to follow up**	0	0.0	-	-	-	2	25.0	-	-	-	N/A

Note: Bold *p*-values indicate statistical significance.

IST, immunosuppressive therapy; PNH, paroxysmal nocturnal haemoglobinuria; +, positive; −, negative; WCC, white cell count; ANC, absolute neutrophil count.

The majority of patients in both groups presented with severe or very severe AA. Two patients without the PNH clone presented with haematuria, but this was not a presenting feature in any patients with the clone. Bleeding from a peripheral site was noted in 70.0% of PNH-positive and 87.5% of PNH-negative patients, while no patients presented with evidence of thromboses. Four PNH-positive (4/10; 40.0%) and four PNH-negative patients (4/8; 50.0%) were given IST. Fifty percent of patients with the PNH clone died, compared to 37.5% among patients without the clone.

Seven of the ten (7/10; 70.00%) PNH-positive patients presented with bleeding (Table 2). Clone sizes in the granulocyte population and monocyte population in each patient ranged between 0.11% and 24.00%. Five of the total PNH-positive patient cohort survived (5/10; 50.00%), four (40.00%) of whom received ATG. The other survivor went on to receive an HSCT and is still alive. The remaining five PNH-positive patients (5/10; 50.00%) died, two from sepsis and three from bleeding. One of these patients died while awaiting HLA results. Among those who died, the PNH clone sizes ranged from 0.30% to 15.00%.

**TABLE 2:** Paroxysmal nocturnal haemoglobinuria clone sizes and clinical presentation and outcome of patients with aplastic anaemia at the Inkosi Albert Luthuli Central Hospital, Durban, South Africa, September 2013 – January 2018.

PNH+ patient number	Clone size (%)	Presenting symptom	Management	Outcome
1	0.11	Bleeding	Transplant	Alive
2	0.30	Bleeding	Symptomatic	Deceased. No HLA match. Platelet antibodies; therefore, not ATG candidate.
3	0.72	Weakness/pallor	ATG	Alive
4	0.72	Bleeding	Symptomatic	Deceased. Bleeding while awaiting HLA results.
5	1.28	Bleeding	Symptomatic	Deceased. ATG approved but not given due to sepsis.
6	6.00	Bleeding	Symptomatic	Deceased. No HLA match. Platelet refractoriness; therefore, not ATG candidate.
7	6.00	Weakness/pallor	ATG	Alive
8	13.22	Bleeding	ATG	Alive
9	15.00	Bleeding	Symptomatic	Deceased. Sepsis before ATG administration.
10	24.0	Weakness/pallor	ATG	Alive

PNH+, paroxysmal nocturnal haemoglobinuria population-positive; ATG, antithymocyte globulin; HLA, human leukocyte antigen.

**FIGURE 3:**
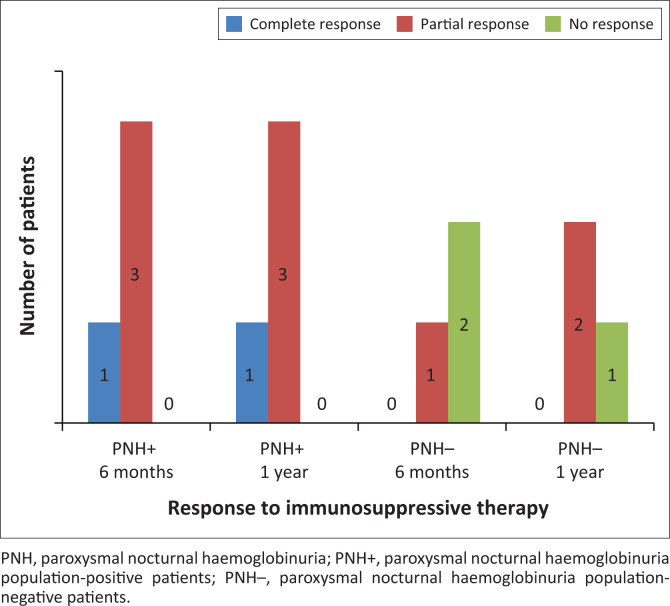
Response to immunosuppressive therapy among paroxysmal nocturnal haemoglobinuria population-positive and paroxysmal nocturnal haemoglobinuria population-negative patients at the Inkosi Albert Luthuli Central Hospital, Durban, South Africa, September 2013 – January 2018.

Eight patients (four PNH-positive and four PNH-negative patients) in our cohort received ATG. All patients received equine ATG, and one patient subsequently received an additional dose of rabbit ATG. One patient in the PNH-negative group died from sepsis a few days after infusion and was excluded from further analysis (Figure 3). At both six and 12 months, one patient in the PNH-positive group had a complete response and the other three patients had a partial response. In the PNH-negative group, one patient had a partial response at six months while two did not respond. At 12 months, the patient with a partial response still had a partial response, one of the patients with no response had developed a partial response, while the other patient remained non-responsive despite receiving a second dose of ATG.

## Discussion

Paroxysmal nocturnal haemoglobinuria is rare in children and does not present classically. The presentation is normally bone marrow failure,^12^ which exists as a continuum with PNH.^10,24^ In this study we found a 55.6% prevalence of PNH clones in those children presenting with AA. The clone size had no impact on the severity of the clinical presentation, but patients appeared to respond well to IST. Those with a PNH clone were also older than their PNH-negative counterparts and had lower total white cell counts.

The differences in the presentation of PNH between adults and children have been described.^8^ A study reported in 1991 from the United States described that haemoglobinuria was detected in 50.0% of adult PNH patients compared to 15.0% among children, and only 25.0% of adult PNH patients had bone marrow failure compared to 58.0% among children.^8^ Paroxysmal nocturnal haemoglobinuria clone sizes have been reported to be much lower in AA than in PNH disease,^25^ requiring high-sensitivity fluorescent aerolysin analysis to detect clones < 0.1%.^26^ No patients in our study presented with clinical PNH, in keeping with a report from Japan also describing PNH clones in AA patients.^2^

The PNH clone prevalence in this study, at 55.6%, is much higher than the 12.9% prevalence observed in a paediatric cohort in India in 2015^27^ that used a PNH clone cut-off of > 1.0%. When a PNH clone size of < 1.0% is included, as in our study, some studies show higher prevalence rates such as 41.0%^28^ and 46.0%.^29^ A study conducted in the United States^30^ found that 40.0% of patients were affected, even at a cut-off of 1.0%. In an adult cohort from Tanzania, a 42.0% prevalence of PNH clones in AA patients was found.^31^ Interestingly, this study also showed an overall higher AA prevalence of six cases per million per year, compared to two per million per year in Europe and North America. A study among a paediatric cohort from Egypt showed the presence of a PNH clone in 36.0% of patients using the CD59 immunohistochemical staining on bone marrow trephine biopsies.^32^

It must be noted that although we included very small clones in our study, the evidence for the impact of these minor clones on clinical presentation is not yet well established.^27^ It has also been shown that small populations of granulocytes bearing the PNH phenotype can even be found in healthy individuals^33^; any specific cut-off value may thus be arbitrary.

The median age of children presenting with a PNH clone was significantly higher (*p* = 0.02). There is also evidence in the literature suggesting that PNH clones increase with age in children.^27,34^ In the current study, children without the PNH clone presented with a higher incidence of haematuria. As all patients presented with thrombocytopenia, the true reason for the haematuria could not be elucidated, particularly because a urine dipstick is unable to distinguish this from haemoglobinuria, the true mark of intravascular haemolysis. Similarly, the median haemoglobin concentration of > 7 g/dL observed in all patients is likely due to blood transfusions received prior to referral. According to Camitta’s criteria for the classification of AA severity (cited by Marsh et al.),^21^ ANC (< 0.5 × 10^9^/L), reticulocyte count (< 20 × 10^9^/L) and platelet count (< 20 × 10^9^/L) are the predictors of severity. The presence of at least two of these three criteria indicates severe AA. A study in Korea highlighted that the ANC should be more heavily weighted, as it has the biggest impact on patient complications, is not influenced by transfusions, and can be readily used to classify severity in patients.^35^ In our cohort, 15 of the 18 patients had a neutrophil count of < 0.5 × 10^9/^L, while the haemoglobin and platelet counts remained above the defined cut-off likely due to transfusions received, suggesting that ANC may be a more important factor to consider in the diagnosis and severity grading of patients with AA.

This study also found no association between PNH clone size and severity of clinical presentation. A study from India published in 2018 also showed no correlation between clone size and PNH symptoms in a large paediatric cohort.^1^ Only three of the 100 patients in the study presented with haemolysis, all of whom had clone sizes > 10%, and one patient with a very small clone presented with thrombosis. This highlights the need for screening all patients for signs of clinical PNH, regardless of clone size.

Importantly, only one child in our cohort received an HSCT. Two patients had already developed platelet refractoriness and this may be due to the continuation of platelet transfusions while awaiting the HLA results. Our findings show an improved ATG response in the PNH-positive cohort; however, the small size of the study population makes it impossible to reach a definitive conclusion. Some studies have either shown, suggested or referenced an improved response to IST in patients with AA presenting with a PNH clone,^18,27,36,37^ or have found no difference in response.^1,28,30^ However, a recent meta-analysis that included 1236 participants from 11 studies showed a statistically significant improved response to IST in patients with PNH clones.^38^ In determining IST response in AA as a whole, the North American Pediatric Aplastic Anemia Consortium published results from 314 paediatric AA patients wherein complete response was observed in 59.8% of patients, and the 5-year event-free survival was 64.0%.^39^ Relapsed or refractory disease is thus still an important factor to consider in the outcomes of these patients, with many either needing a second course of IST or an HSCT. This group found that HSCT in relapsed/refractory disease was the preferred treatment modality, rather than a second course of IST. While HSCT has historically only been the preferred front-line therapy in patients having a matched sibling donor, a recent report by the United Kingdom Paediatric BMT Working Party, Paediatric Diseases Working Party and Severe Aplastic Anaemia Working Party of the European Society for Blood and Marrow Transplantation showed that using a matched unrelated-donor upfront had similar results to matched sibling donors.^40^ Importantly, patients with an upfront-matched unrelated-donor HSCT had superior outcomes than patients who initially failed IST and then went on to receive a matched unrelated-donor HSCT.

Regarding the timing of IST administration, it is important to note that in South Africa, the genetic diversity of the population is such that less than 20.0% of patients (non-European) will have an HLA match for HSCT.^41,42^ Although the paucity of African donors is certainly a problem, the diversity within the HLA region is significant, even among typed donors.^20,43^ Once abnormal cytogenetics, inherited bone marrow failure syndromes and malignancies have been excluded, early initiation of IST is recommended as a delay may lead to increased morbidity and risk of mortality. Some studies suggest that the presence of a PNH clone in and of itself rules out an inherited bone marrow failure syndrome.^1,29^ This may further aid the timeous initiation of therapy.

### Limitations

The lower limit of quantitation or detection for PNH clones has not been defined by our laboratory. The quantitative values below 1.0% must thus be interpreted cautiously as the external quality assurance samples analysed in the laboratory have clone sizes greater than 1.0%. However, the external quality assurance programme used for minimal residual disease monitoring verifies the accuracy of the laboratory in defining clone sizes below 0.1% and the identification of very small clones in patients with bone marrow failure has been consistently reported in the literature. For this reason, we have opted to include all clones. The sample size is small; however, this is attributable to the rarity of PNH in the paediatric population and the lack of testing in all paediatric patients with AA. It is thus not possible to make any definitive conclusions regarding any observed differences between the two populations. Additionally, the PNH-negative group was not monitored for the subsequent development of a PNH clone and the clone sizes in PNH-positive patients were not monitored and correlated to clinical outcomes. With clear evidence of the risk of developing clinical PNH in future,^2^ these are important factors to consider in the future management of these patients.

### Conclusion

Our study found a very high prevalence of PNH clones in patients with AA. Patients with PNH clones were older at presentation and had significantly lower total white cell counts. A higher clone size was not associated with a worse clinical presentation. Patients with PNH clones who received IST survived. In similar settings where significant HLA diversity exists and in the absence of a potential sibling donor, once malignancies and inherited bone marrow failure syndromes have been excluded, patients presenting with AA should be initiated on IST even while awaiting HLA typing results. Larger studies are needed to further determine the impact of PNH clones on the disease course in paediatric patients with AA.
